# Assessing smoking status in disadvantaged populations: is computer administered self report an accurate and acceptable measure?

**DOI:** 10.1186/1471-2288-11-153

**Published:** 2011-11-21

**Authors:** Jamie Bryant, Billie Bonevski, Christine Paul, Christophe Lecathelinais

**Affiliations:** 1 Priority Research Centre for Health Behaviour, University of Newcastle, Hunter Medical Research Institute. Room 230A, Level 2, David Maddison Building, Callaghan NSW 2308 Australia; 2 Health Behaviour Research Group, Priority Research Centre for Health Behaviour, School of Medicine & Public Health, University of Newcastle, Hunter Medical Research Institute. Room 268 Level 2, David Maddison Building, Callaghan NSW 2308 Australia

**Keywords:** Smoking, biochemical validation, carbon monoxide, touch screen computer, acceptability, accuracy

## Abstract

**Background:**

Self report of smoking status is potentially unreliable in certain situations and in high-risk populations. This study aimed to determine the accuracy and acceptability of computer administered self-report of smoking status among a low socioeconomic (SES) population.

**Methods:**

Clients attending a community service organisation for welfare support were invited to complete a cross-sectional touch screen computer health survey. Following survey completion, participants were invited to provide a breath sample to measure exposure to tobacco smoke in expired air. Sensitivity, specificity, positive predictive value and negative predictive value were calculated.

**Results:**

Three hundred and eighty three participants completed the health survey, and 330 (86%) provided a breath sample. Of participants included in the validation analysis, 59% reported being a daily or occasional smoker. Sensitivity was 94.4% and specificity 92.8%. The positive and negative predictive values were 94.9% and 92.0% respectively. The majority of participants reported that the touch screen survey was both enjoyable (79%) and easy (88%) to complete.

**Conclusions:**

Computer administered self report is both acceptable and accurate as a method of assessing smoking status among low SES smokers in a community setting. Routine collection of health information using touch-screen computer has the potential to identify smokers and increase provision of support and referral in the community setting.

## Background

Accurate assessment of smoking status is crucial not only for monitoring smoking prevalence, but also for assessing the effectiveness of smoking cessation interventions. Meta-analysis has shown that the accuracy of self-reported smoking status is high when assessed in the general population, particularly in community settings [1]. However self report tends to be compromised during smoking cessation trials where social desirability bias may influence self report, and among particular population groups where smoking is seen as undesirable, including among pregnant women [2-5], and among individuals with smoking related medical conditions including respiratory diseases [6,7] and cancer [8]. It has therefore been recommended that smoking status be validated using a biochemical marker in certain circumstances including when assessing smoking status in special populations and in situations where contextual demand characteristics may influence accurate reporting [9].

As a result of a comprehensive population based approach to tobacco control, smoking rates in Australia have declined from 28.4% in 1989-1990 [10] to less than 17% in 2007 [11]. While Australia now has one of the lowest smoking rates in the developed world, rates remain significantly high among some disadvantaged sub-groups of the community [12]. For example compared to the whole population smoking prevalence rate of 16.9%, smoking rates reported in the 2007 National Drug Strategy Household survey were 9%-21% higher among disadvantaged sub-groups, including individuals in the lowest socioeconomic quintile (the most disadvantaged; 25.9%), the unemployed (38.2%), and Aboriginal and Torres Strait Islanders (34.1%) [11]. These estimates are however based on self report, the accuracy of which has not been established in highly disadvantaged or very low socio-economic status (SES) populations.

It is important to establish the accuracy of self-report as a measure of smoking status among very low SES populations for a number of reasons, including examining whether social desirability bias may be more or less evident among low SES groups than it is for the general population. Individuals receiving government welfare or community social support may perceive a level of disapproval from others if such support is spent on tobacco products, thereby increasing the likelihood of falsely reporting to be a non-smoker. Alternatively, the greater prevalence of smoking in low SES groups, as well as social norms conducive to smoking, may reduce such social desirability bias. In the absence of relevant data, it is difficult to know whether self-report data for disadvantaged populations provide overestimates or underestimates of the true prevalence of smoking in this population.

One method of assessing smoking status is using touch-screen computer technology. Touch-screen computers are an efficient and cost-effective way of collecting health information, often preferred over pen-and-paper methods [13]. Touch screen computers have been found to be acceptable in a wide range of settings and population groups, including among patients in cancer treatment and rheumatology clinics [14,15], clients of community drug and alcohol treatment centres [16], and in general practice [17]. While the use of touch screen computers has been found to be acceptable among low income populations in primary care [18], no studies have explored the accuracy or acceptability of computer technology for assessing smoking status in a non-health community setting.

This study aimed to determine the accuracy (i.e. sensitivity, specificity, positive predictive value and negative predictive value) and acceptability of computer administered self report of smoking among socially disadvantaged individuals accessing a social and community service organisation (SCSO) for welfare support.

## Materials and methods

### Design

Data were collected as part of a larger cross-sectional health survey. Data collection occurred between February and October 2010.

### Setting & Sample

One SCSO in New South Wales, Australia, participated. Data was collected from three SCSO service sites located in Sydney (two services) and a regional area (one service). SCSOs are non-government, not-for-profit organisations that provide welfare services to highly disadvantaged individuals in the communities in which they are based. They provide a range of services to individuals including financial and family counselling, temporary accommodation, food and material aid, and child and family support [19,20]. Participants were adult clients attending the SCSO for emergency relief, which involved receiving financial or material assistance, including free grocery items, assistance paying bills, and assistance with purchasing medications.

### Recruitment & Procedure

Service attendees were invited by their caseworker at the end of their emergency relief interview to complete a touch screen computer administered health survey. Clients attending the services during the recruitment period who were aged over 18 years, able to speak or read English to a level that allowed completion of an English survey with or without assistance, and who were not distressed were eligible to participate. The gender and date of birth of non-consenting clients were collected to assess participation bias. Clients who consented to participate were introduced to a research assistant who provided support to read and/or complete the survey as necessary. Following completion of the touch screen computer health survey, participants were asked to complete a pen-and-paper survey to determine the acceptability of using the touch screen computer. Participants were then asked to provide a breath sample to measure breath carbon monoxide (BCO). BCO is a portable, low cost, immediate and non-invasive method of assessing smoking status [21], shown to have acceptable sensitivity and specificity [22]. Participants were unaware that they would be asked to provide this sample prior to completing the health survey.

### Measures

#### Self-report

Survey items included questions about social demographics (e.g. gender, age, income, Aboriginal and Torres Strait islander status, employment and education), fruit and vegetable consumption, sun protection practices, smoking, physical activity, alcohol consumption and cancer screening behaviours (see Additional File 1). Only results relevant to the validation of smoking status will be reported here. All participants were asked "Do you currently smoke tobacco products?" (response options: 'Yes, daily', 'Yes, at least once a week', 'Yes, but less often than once per week' and 'No, not at all'). Time since last cigarette was determined by asking "When was the last time you smoked a cigarette, cigar or pipe?" (response options: 'Less than 4 hours ago', 'Between 4 and 8 hours ago', 'Between 8 and 12 hours ago and 'Longer than 12 hours ago'). In order to examine discrepancies between self reported smoking status and BCO, exposure to passive smoke and heaviness of smoking (using the Heaviness of Smoking Index (HSI)) [23] were examined as explanatory factors. All participants were asked "In the last 24 hours have you been near other people who were smoking?" (response options: 'Yes' and 'No). To enable the calculation of the HSI, smokers were also asked "On an average day, how many cigarettes do you smoke?" and "How soon after waking up do you smoke? (response options: 'Within 5 minutes', '6-30 minutes', 31-60 minutes' and 'After 60 minutes).

#### Touch screen computer

All questions were presented on a touch screen computer using Digivey survey software [24]. The touch screen computer was a Dell Latitude XT2 (1.4 GHz processor).

#### BCO

Exhaled BCO measurements were obtained using a Bedfont Micro+™ Smokerlyzer^® ^(Bedfont Scientific, UK, http://www.bedfont.com). Participants were asked to take a deep breath and hold for 15 seconds before exhaling slowly into the smokerlyzer. BCO monitors used in the study were calibrated by the manufacturer before the study commenced. A cut point of 6 parts per million (ppm) was used as recommended by the manufacturer to distinguish between smokers and non-smokers [25].

#### Acceptability

Acceptability of touch screen computer use was assessed using six questions answered on a five point Likert scale from 'Strongly agree' to 'Strongly disagree'. Items included "Completing the survey using the touch screen computer was enjoyable", "Completing the survey using the touch screen computer was easy", "Completing the survey using the touch screen computer was complicated", "Completing the survey using the touch screen computer was stressful", "I would be happy to complete a short survey about my health a few times a year when I came into [community service organisation]" and "I would prefer to answer this survey using a pen-and-paper survey".

### Power calculation

Based on known smoking rates among groups that utilise social and community service organisations [19], it was assumed that approximately 50% of clients attending the service would be smokers. Based on this assumption, and a minimum required sensitivity and specificity of 80%, a sample of 300 participants would allow estimation of sensitivity and specificity of self-report versus BCO with 95% confidence intervals within 6.4% of the point estimate.

### Statistical Analysis

Basic frequencies were calculated and Chi-square tests and Fisher's exact tests used as appropriate to explore differences between groups. Self-reported smoking status was compared to the established cut point (6 ppm) to determine the sensitivity, specificity, and positive and negative predictive values of self-report against BCO, using BCO as the criterion measure. Due to the known short half life of BCO, only individuals reporting daily or occasional smoking who indicated they had smoked a cigarette in the preceding 12 hours were included in the sensitivity and specificity analysis. The HSI was calculated by assigning a value of 0 for those reporting smoking between 0-10 cigarettes per day (CPD), 1 for those reporting 11-20 CPD, 2 for those reporting 21-30 CPD and 3 for those reporting 31 or more CPD. Responses to "How soon after waking up do you smoke?" were assigned values of 0 for those reporting > 60 minutes, 1 for those reporting 31-60 minutes, 2 for those reporting 6-30 minutes and 3 for those reporting < 5 minutes. These two values were then summed to give a score with a range of 0 (low dependence) to 6 (high dependence).

### Ethics Approval

This study was approved by the University of Newcastle Human Research Ethics Committee.

## Results

### Study Sample

**Figure 1:**
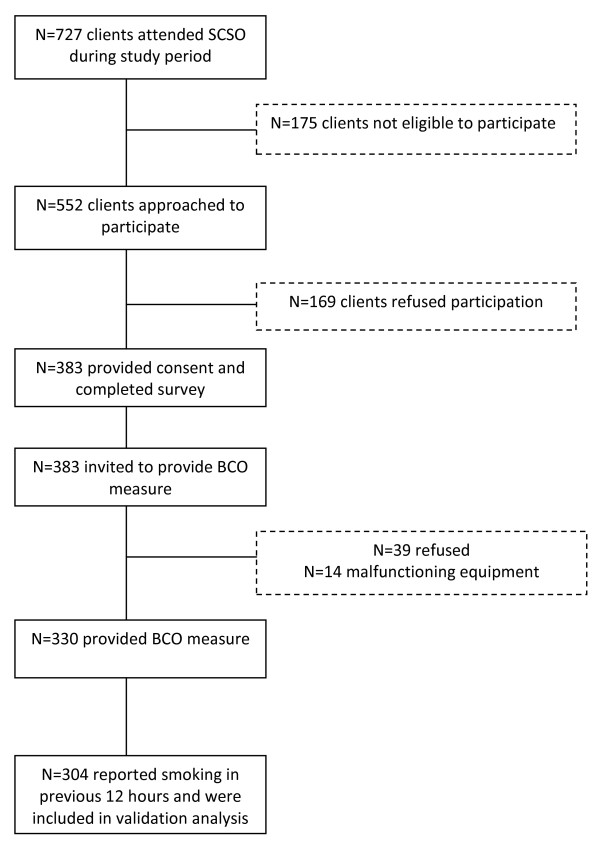
**Participant flow diagram**.

A participant flow diagram is provided in Figure 1. A total of 727 clients attended the three sites during the study period of which 552 were approached to participate. The main reasons for not being approached to participate included having already completed the survey at an earlier visit (71 clients), being assessed by service staff as not suitable to participate (e.g. distressed, unwell, intoxicated or uncooperative, 39 clients), and not being able to speak or read English (13 clients). In total, 383 clients completed the touch screen survey (69% consent rate), of which 330 clients (86%) also provided a breath sample. Demographic details of the sample (n = 330) are presented in table one. Fifty-four percent of participants reported an income of less than AUD$300 per week, 49% were unemployed, 3% reported primary school as their highest level of education and 65% reported secondary school as their highest level of education. Male participants were more likely than female participants to agree to participate (76% vs. 67% respectively, χ^2 ^= 5.5, p = 0.02), and participants recruited from the two inner-city services were more likely to agree to participate than participants from the regional service (80% inner-city vs. 60% regional, χ^2 ^= 34, p < 0.001). A total of 39 clients refused to provide a breath sample and a further 14 clients could not provide a breath sample due to malfunctioning equipment. There were no statistically significant differences in gender, age, Aboriginal or Torres Strait Islander status, marital status, education, income, employment characteristics or smoking status between those consenting and those not consenting to provide a breath sample (see table 1).

**Table 1:** Demographic characteristics and smoking status of whole sample (n = 330) and participants not consenting to provide a breath tests (n = 39)

	Validation sample (n = 330)		Participants not consenting to breath test (n = 39)		**χ**^**2**^
	**N**	**%**	**N**	**%**	

**Gender**					
Male	186	56	17	44	(χ^2 ^= 1.79, *p *= 0.18)
Female	144	44	22	56	

**Age**					
≤ 29 years	45	14	5	13	(χ^2 ^= 1.64, df = 5, *p *= 0.90)
30-39 years	85	26	10	26	
40-49 years	96	29	10	26	
50-59 years	67	20	11	28	
60-69 years	21	6	2	5	
70 + years	16	5	1	3	

**Aboriginal or Torres Strait Islander**					
Yes	39	12	3	8	(χ^2 ^= 0.51, *p *= 0.47)
No	291	88	36	92	

**Marital status**					
Never married/single	178	54	22	56	(χ^2 ^= 0.36, df = 4, *p *= 0.46)
Married	24	7	5	13	
De facto/living with partner	26	8	1	3	
Divorced/separated	80	24	10	26	
Widowed	22	7	1	3	

**Highest level of education**					
Primary school	10	3	1	3	(χ^2 ^= 1.62, df = 4, *p *= 0.8)
High school years 7-10	157	48	15	38	
High school years 11-12	58	17	7	18	
TAFE	56	17	8	21	
University Degree	49	15	8	21	

**Income**					
< $200	53	16	5	13	(χ^2 ^= 7.4, df = 5, *p *= 0.19)
$200-$300	124	38	10	26	
$300-$400	83	25	12	31	
$400-$500	31	9	2	5	
< $500	19	6	6	15	
Prefer not to answer	20	6	4	10	

**Employment**					(χ^2 ^= 6.8, df = 7, *p *= 0.45)
Full time	4	1	0	0	
Part time/casual	25	8	0	0	
Unemployed	162	49	19	49	
Student	15	5	3	8	
Unable to work	15	5	7	18	
Home duties	36	11	3	8	
Retired	38	12	3	8	
Other	35	11	4	10	

**Smoking Status**					
Daily	181	55	17	44	(χ^2 ^= 1.9, df = 3, *p *= 0.6)
Occasional- weekly	13	4	2	5	
Occasional- monthly	11	3	2	5	
Non-smoker	125	38	18	46	

* Note: not all percentages add to 100% due to rounding

### Self-reported smoking status

Of the clients included in the validation analysis (n = 304), 59% (n = 179) reported daily or occasional smoking (at least once per week or once per month). A total of 41% of clients (n = 125) reported being current non-smokers.

### Accuracy of self reported smoking status vs. BCO

The smoking characteristics of participants included in validation analysis are reported in table 2. Self reported daily or occasional smokers (n = 179) had a BCO reading greater than or equal to 6 ppm indicating a sensitivity of 94.4% (CI 91.1%-97.8%). One hundred and sixteen self reported non-smokers had a BCO reading below 6 ppm indicating a specificity of 92.8% (CI 88.3%-97.3%). The positive predictive value was 94.9% and the negative predictive value was 92.0%. Nine participants (3% of the total sample) self reported being non-smokers but returned a BCO reading at or above the 6 ppm cut point. Ten self reported daily or occasional smokers (3.3% of the total sample) returned a BCO below the 6 ppm cut point. Heaviness of Smoking Index and exposure to passive smoke were analysed as explanatory variables for participants whose self reported smoking status and BCO measured smoking status were disparate. Analysis using Fisher's exact revealed no differences in misclassification according to HSI (p = 0.12) or exposure to environmental smoke (p = 0.57).

**Table 2:** Smoking characteristics of participants included in validation analysis (n = 304).

	Male	Female	Total	
	**n**	**n**	**n**	**%**

**Self reported smoking status**				
Smoker- daily or occasional	108	71	179	59
Non-smoker	59	66	125	41
Time since last cigarette*				
< 4 hours	99	66	165	92
4-8 hours	8	5	13	7
8-12 hours	1	0	1	1
**Exposure to passive smoke in last 24 hours**				
Exposure	138	99	237	78
No exposure	28	38	66	22
Missing	1	0	1	0.3
Heaviness of smoking index*				
1-2 (Low dependence)	39	26	65	36
3-4	45	35	80	45
5-6 (High dependence)	24	10	34	19

* Smokers only. n = 179. Note: not all percentages add to 100% due to rounding.

### Touch screen computer acceptability

Acceptability of touch screen computer use is reported in table 3. The majority of participants agreed or strongly agreed that completing the touch screen computer was easy (88%) and enjoyable (79%), and disagreed or strongly disagreed that completing the survey was stressful (92%) or complicated (90%). Most participants (89%) agreed or strongly agreed that they would be happy to complete a survey about their health a few times per year. Only 19% of participants agreed or strongly agreed they would prefer to complete the survey using a pen-and-paper survey.

**Table 3:** Acceptability (%) of touch screen computer use (N = 330)

	Strongly agree	Agree	Neither agree or disagree	Disagree	Strongly disagree
Completing the survey using the touch screen computer was enjoyable	17	62	17	4	0
Completing the survey using the touch screen computer was easy	25	63	10	2	0
Completing the survey using the touch screen computer was complicated	0	4	5	67	23
Completing the survey using the touch screen computer was stressful	0	3	5	62	30
I would be happy to complete a short survey about my health a few times a year when I came into [service]	22	67	9	2	0
I would prefer to answer this survey using a pen-and-paper survey	5	13	24	40	17

^ Note: not all rows sum to 100% due to rounding

## Discussion

Because misreport often occurs when an individual fears disapproval regarding disclosure of smoking status [1], emphasis has been placed on confirming self report of smoking status using biochemical measures in high-risk population groups. Little work has examined the accuracy of self reported smoking among highly disadvantaged smokers who are often heavily nicotine dependent and live in communities with high smoking rates and pro-smoking social norms. This study aimed to assess the acceptability and accuracy of computer administered self-report of smoking among a low SES population attending a social and community welfare organisation.

Our findings indicate a strong agreement between self reported smoking status and BCO measured smoking status, with just over 6% of participants (an equal number of self reported smokers and non-smokers) misclassified by self report. This was significantly lower than levels of misreport found among other population groups, including pregnant Indigenous women [26]. No correlation was found between reports of being exposed to passive smoke or heaviness of smoking and misclassification, suggesting these smokers were misreporting their smoking status. These findings suggest that self-report is likely to be valid in determining smoking status in low SES community based populations.

The sensitivity and specificity for self-reported smoking against BCO at 94.4% sensitivity and 92.8% specificity are higher than mean figures derived in a review of validation studies using BCO in general community samples (87% sensitivity, 89% specificity [1]. A sensitivity analysis conducted using Receiver Operating Curve analysis (results not reported) found that by lowering the cut-point to 5 ppm, sensitivity and specificity further improved (96.7% and 91.2% respectively), and resulted in a greater percentage of participants being correctly classified (94.4%) compared to our cut point of 6 ppm (93.6% correctly classified). Other published research has found that cut-points lower than those recommended are optimal for certain sub-groups [27-29]. Future clinical research using BCO for monitoring or feedback should further explore optimal cut points, as well as determine the accuracy of self report among low SES individuals in high-demand situations, such as during smoking cessation trials.

The high level of acceptability of touch screen computer use in this population supports research demonstrating the utility of touch screen technology as an efficient method of routinely collecting information in health care settings [16,17,30]. Participants rated the touch screen computer as easy to use and enjoyable, and agreed they would be happy to complete a similar survey a few times each year. Given the high degree of acceptability, the potential for integrating the routine collection of health risk information into SCSOs should be further explored. These organisations are well placed to provide advice and referral regarding health care needs to the large number of socially disadvantaged clients seen for welfare and social support. Collection of health care information via touch screen computer may provide an efficient way of identifying those smokers and providing assistance with social and health care needs simultaneously.

The high consent rate for BCO testing (86%) also indicates very good acceptability of BCO among clients attending the SCSO. It was the experience of the authors that the immediate return of results to clients often started conversations about smoking and quitting, suggesting a potential role for BCO as a clinical tool to educate and motivate low SES smokers who are not motivated to quit. While there is currently no strong evidence that biofeedback increases cessation attempts [31], BCO may be an acceptable and non-threatening way to engage hard-to-reach groups with smoking cessation and prompt advice and referral, especially given the high prevalence of smoking identified in this setting.

## Limitations and Generalisability

As participants were not told that their smoking status would be verified prior to self report of smoking status, these results may not be generalisable to situations where individuals are aware that the accuracy of their report will be confirmed. The limitations of BCO as a biochemical confirmer of smoking status should also be recognised. Because BCO is a short-term measure of exposure to tobacco smoke, with a half life of 2-8 hours [9], it is possible that self-reported smokers who had consumed their last cigarette longer than within 2-8 hours of providing a breath sample may have been incorrectly classified by BCO as non-smokers. To control for the short half life, we included in the sensitivity analysis only the smokers who reported smoking their last cigarette within the preceding 12 hours. Further, compared with other biochemical measures of confirming smoking status such as cotinine, BCO may not detect very low levels of smoking and can be influenced by environmental sources of CO [9]. However these limitations are outweighed by the practical advantages of using BCO which is an immediate, low-cost and portable measure of confirmation.

## Conclusions

Computer administered self report is an accurate and acceptable method of assessing smoking status in a low SES sample of smokers in a community setting, with a low rate of misclassification identified. Routine collection of health information via touch screen computer holds potential as a way to improve the health of low SES individuals attending community welfare organisations.

## Competing interests

The authors declare that they have no competing interests.

## Authors' contributions

JB, BB, and CP conceived of the study and were involved in its design and co-ordination. JB and BB supervised data collection. Statistical analysis was carried out by JB and CL. JB led manuscript preparation. All authors were involved in data interpretation and revised the manuscript critically for intellectual content. All authors approve of the final version of the manuscript.

## Pre-publication history

The pre-publication history for this paper can be accessed here:

http://www.biomedcentral.com/1471-2288/11/153/prepub

## Supplementary Material

Additional file 1 **Survey Items**. Survey items completed by participants. Click here for file
