# Supplementary material for: Assessing smoking status in disadvantaged populations: is computer administered self report an accurate and acceptable measure?
Source: BMC Med Res Methodol. 2011 Nov 21;11:153. doi: 10.1186/1471-2288-11-153 (PMC3233509; doi:10.1186/1471-2288-11-153)
Supplement: Additional file 1 — Survey Items. Survey items completed by participants. [file 1471-2288-11-153-S1.DOC]

First, we would like to know a little bit about you.

1. Are you

| Male | 1 |
| --- | --- |
| Female | 2 |

1. In what year were you born?

1

9

1. Are you of Aboriginal or Torres Strait Islander origin?

| Yes | 1 |
| --- | --- |
| No | 2 |

1. What is your marital status?

| Married | 1 |
| --- | --- |
| Defacto or living with a partner | 2 |
| Separated or divorced | 3 |
| Never married or single | 4 |
| Widowed | 5 |

1. What is the highest level of education that you have completed?

| Primary school | 1 |
| --- | --- |
| High school years 7-10 | 2 |
| High school years 11-12 | 3 |
| TAFE | 4 |
| University Degree | 5 |

1. What is your household income each week?

| Less than $100 per week | 1 |
| --- | --- |
| Between $100 -$200 per week | 2 |
| Between $200-$300 per week | 3 |
| Between $300-$400 per week | 4 |
| Between $400-500 per week | 5 |
| More than $500 per week | 6 |
| Prefer not to answer | 7 |

1. How would you best describe your employment situation at the moment?

| Employed full time | 1 |
| --- | --- |
| Employed part time or casual | 2 |
| Unemployed | 3 |
| Student | 4 |
| Retired | 5 |
| Permanently unable to work | 6 |
| Home duties | 7 |
| Other | 8 |

1. What is the postcode of the suburb where you live? If you don’t know the postcode, please type ‘0000’. Press CLR if you make a mistake
2. How many serves of vegetables do you usually eat each day? A ‘serve’ is ½ cup of cooked vegetables like carrot or peas, or 1 cup of salad

| 1 serve per day | 1 | GO TO Q12 |
| --- | --- | --- |
| 2 serves per day | 2 | GO TO Q12 |
| 3 serves per day | 3 | GO TO Q12 |
| 4 serves per day | 4 | GO TO Q12 |
| 5 or more serves per day | 5 | GO TO Q12 |
| I don’t eat vegetables every day | 6 | GO TO Q10 |

1. How many serves would you usually eat PER WEEK? If you do not eat *vegetables at least weekly, please type ‘0’.*

serves per week

1. What is the main reason you do not eat vegetables every day? Choose as many options as apply

| I don’t like vegetables | 1 |
| --- | --- |
| Vegetables are too expensive to buy | 2 |
| There are few places to buy vegetables where I live | 3 |
| I don’t have time to cook vegetables | 4 |
| I don’t know how to cook vegetables | 5 |
| I forget to eat vegetables | 6 |
| I don’t have the cooking equipment to prepare vegetables | 7 |
| Other reason | 8 |

1. How many serves of fruit do you usually eat EACH DAY? A ‘serve’ is 1 medium piece of fruit like an apple, 2 small pieces like apricots or 1 cup of chopped or canned fruit

| 1 serve per day | 1 | GO TO Q15 |
| --- | --- | --- |
| 2 serves per day | 2 | GO TO Q15 |
| 3 serves per day | 3 | GO TO Q15 |
| 4 serves per day | 4 | GO TO Q15 |
| 5 or more serves per day | 5 | GO TO Q15 |
| I don’t eat fruit every day | 6 | GO TO Q13 |

1. If you don’t eat fruit every day, how many serves do you eat PER WEEK? If you do not eat *vegetables at least weekly, please type ‘0’.*

serves per week

1. What is the main reason you do not eat fruit every day?

| I don’t like fruit | 1 |
| --- | --- |
| Fruit is too expensive to buy | 2 |
| There are few places to buy fruit where I live | 3 |
| I forget to eat fruit | 4 |
| Other | 5 |

1. In the last week, HOW MANY times have you walked continuously, for at least 10 minutes, for recreation, exercise or to get to or from places?

□□ times

1. What do you estimate was the TOTAL TIME you spent walking in this way in the last week? Please answer in MINUTES

□□ minutes

1. In the last week, HOW MANY times did you do any more moderate physical activities like gentle swimming, social tennis or golf?

□□ times

1. What do you estimate was the TOTAL TIME you spent doing these moderate activities in the last week? Please answer in MINUTES

□□□ minutes

1. In the last week, HOW MANY times did you do any vigorous physical activity which made you breathe harder or puff and pant, like jogging, cycling, aerobics or competitive tennis?

□□ times

1. What do you estimate was the TOTAL TIME you spent doing this vigorous physical activity in the last week? Please answer in MINUTES

□□□ minutes

1. When you are outside for more than 15 minutes on a summer day, how often do you wear a hat to protect yourself from the sun?

| **Never** | **Rarely** | **Sometimes** | **Usually** | **Always** |
| --- | --- | --- | --- | --- |
| 1 | 2 | 3 | 4 | 5 |

1. When you are outside for more than 15 minutes on a summer day, how often do you wear sunglasses to protect yourself from the sun?

| **Never** | **Rarely** | **Sometimes** | **Usually** | **Always** |
| --- | --- | --- | --- | --- |
| 1 | 2 | 3 | 4 | 5 |

1. When you are outside for more than 15 minutes on a summer day, how often do you apply sunscreen to protect yourself from the sun?

| **Never** | **Rarely** | **Sometimes** | **Usually** | **Always** |
| --- | --- | --- | --- | --- |
| 1 | 2 | 3 | 4 | 5 |

1. When you are outside for more than 15 minutes on a summer day, how often do you wear clothing like long sleeves or long pants to protect yourself from the sun?

| **Never** | **Rarely** | **Sometimes** | **Usually** | **Always** |
| --- | --- | --- | --- | --- |
| 1 | 2 | 3 | 4 | 5 |

1. When you are outside for more than 15 minutes on a summer day, how often do you stay in the shade when outdoors to protect yourself from the sun?

| **Never** | **Rarely** | **Sometimes** | **Usually** | **Always** |
| --- | --- | --- | --- | --- |
| 1 | 2 | 3 | 4 | 5 |

1. Do you currently smoke tobacco products?

| Yes, Daily | 1 | GO TO Q27 |
| --- | --- | --- |
| Yes, At least once a week | 2 | GO TO Q27 |
| Yes, but less often than once a week | 3 | GO TO Q27 |
| No, Not at all | 4 | GO TO Q28 |

1. When was the last time you smoked a cigarette, cigar or pipe?

| Less than 4 hours ago | 1 | GO TO Q30 |
| --- | --- | --- |
| Between 4 and 8 hours ago | 2 | GO TO Q30 |
| Between 8 and 12 hours ago | 3 | GO TO Q30 |
| Longer than 12 hours ago | 4 | GO TO Q30 |

1. Have you ever been a daily smoker?

| Yes | 1 | GO TO Q49 |
| --- | --- | --- |
| No | 2 | GO TO Q45 |

1. How long has it been since you quit smoking?

| Less than 6 months | 1 |
| --- | --- |
| Between 6 and 12 months | 2 |
| Between 1 and 2 years | 3 |
| Between 2 and 5 years | 4 |
| More than 5 years | 5 |

1. What type of tobacco do you normally use? Choose as many answers as apply

| Cigarettes (Pre-rolled) | 1 |
| --- | --- |
| Cigarettes (Roll your own) | 2 |
| Cigars or Pipe | 3 |
| Chewing tobacco | 4 |
| Chop chop | 5 |
| Snuff | 6 |

1. On an average day, how many cigarettes do you smoke? Press CLR if you make a mistake
2. How much do you spend on average on tobacco each week? Enter your answer in dollars ($). Press CLR if you make a mistake
3. In the last six months have you spent money on cigarettes that you knew would be better spent on household essentials like food?

| Yes | 1 |
| --- | --- |
| No | 2 |

1. At what age did you first start smoking daily?

□□ years

1. How soon after waking up do you smoke?

| Within 5 minutes | 1 |
| --- | --- |
| 6-30 minutes | 2 |
| 31-60 minutes | 3 |
| After 60 minutes | 4 |

1. Have you ever tried to quit smoking before?

| Yes | 1 | GO TO Q37 |
| --- | --- | --- |
| No | 2 | GO TO Q39 |

1. How many times have you made a quit attempt that lasted at least one day in the past 12 months? Press CLR if you make a mistake
2. What methods have you used when trying and quit smoking in the past? Choose as many answers as apply.

| I had no help (quit cold turkey) | 1 |
| --- | --- |
| I contacted the Quitline | 2 |
| I used nicotine replacement therapy (patches, gum, inhaler) | 3 |
| I received support from family or friends | 4 |
| I had individual quit smoking counselling | 5 |
| I joined a quit smoking group program | 6 |
| I used acupuncture or hypnosis | 7 |
| Other | 8 |

1. What persons have advised you to quit in the last 12 months? Choose as many answers as apply

| No one has advised me to quit in the last 12 months | 1 |
| --- | --- |
| Doctor | 2 |
| Nurse | 3 |
| Family member | 4 |
| Friend | 5 |
| Caseworker | 6 |
| Teacher | 7 |
| Boss at work | 8 |
| Other person | 9 |

1. Which statement best describes how interested you are in quitting smoking?

| I am not interested in quitting smoking | 1 |
| --- | --- |
| I am a little bit interested in quitting smoking | 2 |
| I am quite interested in quitting smoking | 3 |
| I am very interested in quitting smoking | 4 |

1. What are your intentions regarding quitting? Do you plan to

| Quit in the next 30 days | 1 |
| --- | --- |
| Quit in the next 6 months | 2 |
| Quit, but not in the next 6 months | 3 |
| Never quit | 4 |
| Don’t know | 5 |

1. In the last 24 hours have you been near other people who were smoking?

| Yes | 1 |
| --- | --- |
| No | 2 |

1. Would you be interested in getting help from staff at this service to help you quit smoking?

| Yes | 1 | GO TO Q44 |
| --- | --- | --- |
| No | 2 | GO TO Q45 |

1. What types of help would you like to receive from staff at this service to quit smoking? Select as many answers as apply

| To be asked by staff at this service if I would like help to quit smoking | 1 |
| --- | --- |
| To get support and encouragement from staff to help me quit | 2 |
| To be put in touch with telephone help like Quitline | 3 |
| To be given pamphlets about quitting smoking | 4 |
| To be given free or cheap nicotine patches or gum | 5 |
| To be given one-on-one advice or counselling | 6 |
| To be given cash rewards for quitting and staying quit | 7 |
| To be given non-cash rewards like footy tickets or shop vouchers for quitting | 8 |
| To be given computer or internet based quit programs | 9 |
| To be given a Video or DVD about quitting smoking | 10 |
| To be given an alternative therapy like hypnosis or acupuncture | 11 |
| To be given quit help via SMS messages | 12 |

1. Some people suggest the health system should pay people to improve their health. Do you think that paying people to quit smoking is….

| An excellent idea | A good idea | A bad idea | A very bad idea | Don’t know |
| --- | --- | --- | --- | --- |

1. Do you think that paying people to quit smoking would do more good than harm?

| Strongly agree | Agree | Neither Agree or Disagree | Disagree | Strongly Disagree |
| --- | --- | --- | --- | --- |

1. Do you think that paying people to quit smoking would motivate smokers to quit?

| Strongly agree | Agree | Neither Agree or Disagree | Disagree | Strongly Disagree |
| --- | --- | --- | --- | --- |

1. How much money should the government pay a smoker to quit for 12 months?

| $0 | 1 |
| --- | --- |
| $50 | 2 |
| $100 | 4 |
| $250 | 5 |
| $1000 | 6 |
| $1500 | 7 |
| More than $1500 | 8 |

1. Have you has an alcoholic drink of any kind in the last 12 months?

| Yes | 1 | GO TO Q50 |
| --- | --- | --- |
| No | 2 | GO TO Q53 |

1. How often did you have a drink containing alcohol in the past year?

| Never | 1 |
| --- | --- |
| Monthly or less | 2 |
| 2 to 4 times a month | 3 |
| 2 to 3 times a week | 4 |
| 4 to 5 times a week | 5 |
| 6 or more times a week | 6 |

1. How many standard drinks containing alcohol did you have on a typical day when you were drinking in the past year?

| Never | 1 |
| --- | --- |
| 1 to 2 drinks | 2 |
| 3 to 4 drinks | 3 |
| 5 to 6 drinks | 4 |
| 7 to 9 drinks | 5 |
| 10 or more drinks | 6 |

1. How OFTEN do you have four or more Standard Drinks on one occasion in the past year?

| Never | 1 |
| --- | --- |
| Less than monthly | 3 |
| Monthly | 4 |
| Weekly | 5 |
| Daily or almost daily | 6 |

1. Have you ever had a Pap test? [Note- question asked only if answer to question 1= Female]

| Yes | 1 | GO TO Q54 |
| --- | --- | --- |
| No | 2 | GO TO Q55 |
| Don’t know | 3 | GO TO Q55 |

1. How long ago was your last Pap test?

| Less than 12 months ago | 1 |
| --- | --- |
| Between 12 months and 2 years ago | 2 |
| Between 2 and 3 years ago | 3 |
| Between 3 and 5 years ago | 4 |
| More than 5 years ago | 5 |

1. Have you ever had a screening mammogram? [Note- question asked only if answer to question 1= Female]

| Yes | 1 | GO TO Q56 |
| --- | --- | --- |
| No | 2 | GO TO Q57 |
| Don’t know | 3 | GO TO Q57 |

1. How long ago was your last mammogram?

| Less than 12 months ago | 1 |
| --- | --- |
| Between 12 months and 2 years ago | 2 |
| Between 2 and 3 years ago | 3 |
| Between 3 and 5 years ago | 4 |
| More than 5 years ago | 5 |

1. Have you ever had a Faecal Occult Blood test?

| Yes | 1 | GO TO Q58 |
| --- | --- | --- |
| No | 2 | GO TO Q59 |
| Don’t know | 3 | GO TO Q59 |

1. How long ago was your last faecal occult blood test?

| Less than 12 months ago | 1 |
| --- | --- |
| Between 12 months and 2 years ago | 2 |
| Between 2 and 3 years ago | 3 |
| Between 3 and 5 years ago | 4 |
| More than 5 years ago | 5 |

1. Have you ever had a PSA test? [Note- question asked only if answer to question 1= Male]

| Yes | 1 | GO TO Q60 |
| --- | --- | --- |
| No | 2 | FINISH |
| Don’t know | 3 | FINISH |

1. How long ago was your last PSA test?

| Less than 12 months ago | 1 |
| --- | --- |
| Between 12 months and 2 years ago | 2 |
| Between 2 and 3 years ago | 3 |
| Between 3 and 5 years ago | 4 |
| More than 5 years ago | 5 |

**Thank you**
